# Predicting breast cancer risk using interacting genetic and demographic factors and machine learning

**DOI:** 10.1038/s41598-020-66907-9

**Published:** 2020-07-06

**Authors:** Hamid Behravan, Jaana M. Hartikainen, Maria Tengström, Veli–Matti Kosma, Arto Mannermaa

**Affiliations:** 1 Institute of Clinical Medicine, Pathology and Forensic Medicine, and Translational Cancer Research Area, University of Eastern Finland, P.O. Box 1627, FI-70211 Kuopio, Finland; 2 Biobank of Eastern Finland, Kuopio University Hospital, Kuopio, Finland; 3 Institute of Clinical Medicine, Oncology, University of Eastern Finland, P.O. Box 1627, FI-70211 Kuopio, Finland; 4 Cancer Center, Kuopio University Hospital, Kuopio, P.O. Box 100, FI-70029 Kuopio, Finland

**Keywords:** Cancer genomics, Cancer genetics, Breast cancer, Machine learning

## Abstract

Breast cancer (BC) is a multifactorial disease and the most common cancer in women worldwide. We describe a machine learning approach to identify a combination of interacting genetic variants (SNPs) and demographic risk factors for BC, especially factors related to both familial history (Group 1) and oestrogen metabolism (Group 2), for predicting BC risk. This approach identifies the best combinations of interacting genetic and demographic risk factors that yield the highest BC risk prediction accuracy. In tests on the Kuopio Breast Cancer Project (KBCP) dataset, our approach achieves a mean average precision (mAP) of 77.78 in predicting BC risk by using interacting genetic and Group 1 features, which is better than the mAPs of 74.19 and 73.65 achieved using only Group 1 features and interacting SNPs, respectively. Similarly, using interacting genetic and Group 2 features yields a mAP of 78.00, which outperforms the system based on only Group 2 features, which has a mAP of 72.57. Furthermore, the gene interaction maps built from genes associated with SNPs that interact with demographic risk factors indicate important BC-related biological entities, such as angiogenesis, apoptosis and oestrogen-related networks. The results also show that demographic risk factors are individually more important than genetic variants in predicting BC risk.

## Introduction

Automatic cancer risk prediction is the task of discriminating cancer cases from healthy controls by incorporating individual sources of variation, such as demographic and epidemiological information^1^, genomes^2^, transcriptomes^3^, miRNAomes^4^, metabolomes^5^ and clinical data^6^. This task has attracted increasing attention in recent years due to an increase in both the amount and types of collected health-related data as well as advances in computational modelling approaches, which have made the processing of such data feasible. Most existing cancer risk prediction systems are based on individual sources of variation. For example, an ovarian cancer risk prediction model was built in^1^ using epidemiological risk factors, such as the age at menopause, duration of hormone replacement therapy and body mass index. Farina *et al*. evaluated the ability of C-miRNAs to identify women most likely to develop BC by profiling miRNA from serum obtained long before diagnosis^4^. They found 6 miRNA risk signatures that could discriminate high-risk women who develop BC from those who remain cancer-free. Similarly, Dougan *et al*. observed that out of 661 metabolites detected, 24 metabolites differ significantly among BC cases and controls using a feature-by-feature analysis approach^5^. It should be noted that cancer is a multi-factorial disease caused by lifestyle, genetic, and environmental factors^7^. Analysis of individual sources of variations may not be beneficial in creating a comprehensive view of the disease; thus, integrative approaches, which combine different sources of data, are considered necessary for risk evaluation^8^.

In the case of BC, researchers have identified quantifiable BC risk factors, including genetic variants^9^, epidemiological factors^10,11^ and abnormalities observed in mammography screenings^12^, for BC risk evaluation. Although these individual risk factors are important for risk evaluation, little is known about how a combination of multiple risk factors, as predictor variables, can improve BC risk prediction accuracy. For genetic variants, the polygenic risk score (PRS) model aggregates the effects of risk alleles associated with the disease using the effect of multiple SNPs with variable effect sizes obtained from genome-wide association studies (GWASs)^13^. However, the PRS assumes that the disease-associated SNPs are independent of each other and that the risk effects are linear and additive^13^.

Machine learning (ML) approaches have enabled combinations of multiple types of clinical and biological data to make accurate risk predictions^8^. Additive and multiplicative models are two classical ML approaches for modelling the effect of multiple factors on disease. Both approaches are based on regression methods; in additive models, the risk of disease has an additive form that generally uses linear regression, while multiplicative models use logistic regression to report the relative risk or odds ratio (OR)^14^. Using a multiplicative approach, the breast and ovarian analysis of disease incidence and carrier estimation algorithm (BOADICEA) was developed to identify high-risk women based on known genetic and non-genetic risk factors, including information on BC pathology, demographic factors, and variants of high-risk genes^15^. Although the BOADICEA model has been validated with large-cohort data, its discriminatory power in identifying high-risk women is limited^16–18^. The model assumes that risk factors are independent of each other and interact in a linear way with BC development^19^. Feld *et al*.^6^ also evaluated the predictive performance of combinations of 4 demographic risk factors, 10 published BC risk-associated SNPs, and 4 mammography features to predict BC risk in a case-control study with four logistic regression models. They showed that a combination of data improves BC risk prediction over methods that use only a subset of features. However, one should note that these studies are often based on a limited number of predictor variables and conventional regression models, which might make the estimates imprecise when working with potential multicollinearity in high-dimensional medical data, such as in genetic variants^20^. To address this knowledge gap, in this study, we adopt our ML approach previously published in^21^, which is built on an extreme gradient tree boosting (XGBoost) model^22^ followed by adaptive iterative feature selection, to capture optimal networks of interacting features (genetic variants and demographic risk factors for BC) in a BC risk prediction task.

Most previous studies used either a single BC risk factor or a combination of known risk factors, e.g., genetic variants, for BC risk prediction. Recently, we have shown that in a BC risk prediction task, networks of interacting SNPs, identified by a data-driven ML approach, outperform a system based on 51 already known BC-risk-associated SNPs identified by GWASs. In this study, we extend our previous achievements in^21^ by combining networks of interacting genetic variants with demographic risk factors for BC, in the form of risk factors related to both familial history and oestrogen metabolism. Breast cancer risk factors related to familial history, such as having one or more first-degree relatives with BC, are already known to increase the risk of developing BC^23^. Studies have identified a number of genes that are responsible for this inherited increased risk, such as the *BRCA1*, *BRCA2* and *CHEK2* genes^24^. Similarly, factors associated with elevated levels of oestrogen throughout a woman’s lifetime, such as exposure to oestrogen over long periods of time and early onset of menstruation, have been associated with an increased risk of BC^25^.

**Figure 1:**
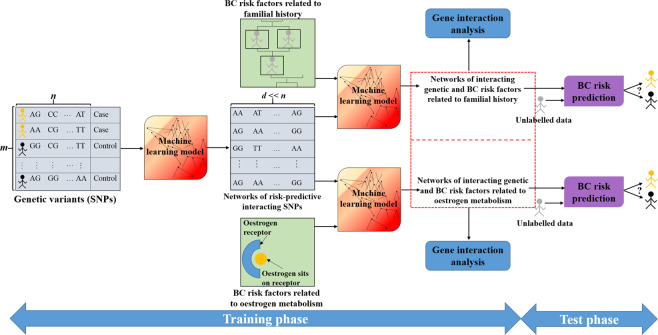
Outline of the proposed BC risk prediction system using ML. In the training phase, networks of interacting genetic and demographic risk factors for BC are identified. These networks of features are then used to predict whether an unlabelled individual is a cancer case or a healthy control in the testing phase. This study provides two examples showing that a combination of interacting genetic variants (SNPs) with BC risk factors related to both familial history and oestrogen metabolism can increase BC risk prediction accuracy.

Figure 1 illustrates the outline of the BC risk prediction system developed in this work. The ML model is trained to find the best groups of interacting genetic and demographic risk factors that contribute to BC risk prediction. We propose that a unified BC risk prediction system that takes advantage of the interactions among both the risk factors within a family of variables (e.g., genetic variants) and the risk factors in different families of variables (e.g., genetic variants and demographic features) is highly desirable in the BC risk evaluation task. Note that this study serves as an example showing how ML can combine different components of cancer risk for risk evaluation, and the proposed approach can be extended to other multifactorial diseases.

We demonstrate our approach on the KBCP dataset^26^, which contains both genotyped data and demographic risk factors in 445 BC cases and 250 controls. We compare our proposed system with analyses based on only demographic risk factors for BC or on genetic variants. We further evaluated our approach against a model that combines 82 known BC-risk-associated SNPs and known demographic risk factors for BC.

## Experimental setup

### Subjects

To perform the BC-risk prediction task, we used the KBCP dataset, in which both genetic variants and demographic features are available for each subject^26^. Genotyping was performed using a custom Illumina array iCOGS with 211,155 SNPs. The genotyping, allele calling, and quality control for the Breast Cancer Association Consortium and iCOGS study are described in detail in Michailidou *et al*.^2^. The samples were obtained with informed written consent. The KBCP dataset, including all methods, was approved by the ethical committee of the University of Eastern Finland and Kuopio University Hospital, and all methods were performed in accordance with the relevant guidelines and regulations. The controls were carefully selected to match each BC case individually by age and long-term area of residence and thus very likely originated from the same genetic background as the cases.

We further selected two groups of known BC risk factors, including 9 available features related to the familial history of BC (Group 1)^23^ and 11 features related to oestrogen metabolism (Group 2)^25^, to combine with the SNP data. Tables 1 and 2, respectively, show the full description of the Group 1 and the Group 2 features and their distributions among the BC cases and controls in the KBCP dataset. Note that there might exist other known or unknown demographic-related BC risk factors that were not considered in this study.

**Table 1:** Distribution of the BC risk factors related to familial history (Group 1) in the KBCP dataset.

Feature names	Cases (N = 445)		Controls (N = 250)	All subjects (N = 695)	Feature description	*p*-value
Cancer in family	**0**	196 (44%)	136 (54%)	332 (48%)	Whether there is any cancer in family members: 0: No; 1: Yes	**0.01**
	**1**	249 (56%)	114 (46%)	363 (52%)		
Cancer type 1	**0**	394 (89%)	235 (94%)	629 (90%)	Type of cancer in the 1st family member with cancer: 0: Other; 1: Breast	**0.02**
	**1**	51 (11%)	15 (6%)	66 (10%)		
Cancer type 2	**0**	437 (98%)	249 (99%)	686 (99%)	Type of cancer in the 2nd family member with cancer: 0: Other; 1: Breast	0.2
	**1**	8 (2%)	1 (1%)	9 (1%)		
First-degree relative 1	**0**	398 (89%)	238 (95%)	636 (91%)	whether the 1st family member with breast cancer is a first-degree relative: 0: No; 1: Yes	**0.01**
	**1**	47 (11%)	12 (5%)	59 (9%)		
First-degree relative 2	**0**	438 (98%)	249 (99%)	687 (99%)	whether the 2nd family member with breast cancer is a first-degree relative: 0: No; 1: Yes	0.3
	**1**	7 (2%)	1 (1%)	8 (1%)		
No. of BCs	**0**	393 (88%)	235 (94%)	628 (90%)	Number of family members with BC.	**0.04**
	**1**	45 (10%)	14 (5%)	59 (9%)		
	**2**	7 (2%)	1 (1%)	8 (1%)		
BC risk score	**0**	396 (89%)	238 (95%)	634 (91%)	Number of first-degree family members with BC.	**0.02**
	**1**	44 (10%)	11 (4%)	55 (8%)		
	**2**	5 (1%)	1 (1%)	6 (1%)		
Lateral 1	**0**	394 (88%)	235 (94%)	629 (90%)	Whether the 1st family member has unilateral or bilateral BC: 0: No tumour; 1: Unilateral; 2: Bilateral	0.06
	**1**	48 (11%)	14 (5%)	62 (9%)		
	**2**	3 (1%)	1 (1%)	4 (1%)		
Lateral 2	**0**	437 (97%)	249 (99%)	686 (97%)	Whether the 2nd family member has unilateral or bilateral BC: 0: No tumour; 1: Unilateral; 2: Bilateral	0.2
	**1**	7 (2%)	1 (1%)	8 (2%)		
	**2**	1 (1%)	0 (0%)	1 (1%)		

The *P*-values denote the differences in the Group 1 features between the BC cases and controls using the chi-squared test for categorical variables. The difference is statistically significant when the *p*-value < 0.05 (highlighted *p*-values). *P*-values were not adjusted for multiple testing.

**Table 2:** Distribution of BC risk factors related to oestrogen metabolism (Group 2) in the KBCP dataset.

Feature names	Cases (N = 445)		Controls (N = 250)	All subjects (N = 695)	Description	*p*-value
Oral contraceptive use	**0**	300 (67%)	124 (49%)	424 (61%)	If subject has used oral contraceptives: 0: No; 1: Yes	**5e-6**
	**1**	145 (33%)	126 (51%)	271 (39%)		
Oral contraceptive use duration	**Mean (std)**	48.27 (51.50)	40.86 (38.91)	44.82 (46.22)	Duration of oral contraceptive use in months.	0.08
Menopausal status	**0**	127 (29%)	109 (44%)	236 (34%)	If menopause has occurred: 0: No; 1: Yes	**8e-5**
	**1**	318 (71%)	141 (56%)	459 (66%)		
Breast- feeding	**0**	107 (24%)	39 (16%)	146 (21%)	Whether the subject has breast-fed: 0: No; 1: Yes	**0.01**
	**1**	338 (76%)	211 (84%)	549 (79%)		
Pregnancy	**0**	85 (19%)	31 (12%)	116 (17%)	Whether the subject has had a full-term pregnancy: 0: No; 1: Yes	**0.03**
	**1**	360 (81%)	219 (88%)	579 (83%)		
Hormonal replacement therapy, oestrogen duration	Mean (std)	53.94 (58.03)	39.85 (47.86)	48.25 (54.59)	Duration of oestrogen use in months.	0.6
Menstrual cycle length	Mean (std)	26.94 (2.27)	27.26 (2.65)	27.06 (2.42)	Length of menstrual cycle in days.	0.09
First pregnancy length	Mean (std)	39.20 (3.37)	39.18 (3.88)	39.19 (3.58)	Length of 1st pregnancy in weeks.	0.12
Second pregnancy length	Mean (std)	39.66 (1.95)	38.99 (4.64)	39.39 (3.31)	Length of 2nd pregnancy in weeks.	**0.03**
Third pregnancy length	Mean (std)	38.97 (3.93)	38.64 (5.70)	38.85 (4.66)	Length of 3rd pregnancy in weeks.	0.12
Body mass index (BMI)	Mean (std)	26.49 (4.60)	26.04 (4.29)	26.33 (4.50)	Body mass index.	0.2

The *P*-values denote the differences in the Group 2 features between the BC cases and controls using the chi-squared test for categorical variables and the *t*-test for continuous variables. The difference is statistically significant when the *p*-value < 0.05 (highlighted *p*-values). *P*-values were not adjusted for multiple testing. std: standard deviation.

## Methods

We adopted the method we developed in^21^ to identify the best combinations of SNPs and demographic features with the highest BC-risk-predictive potential. The ML approach is based on a gradient tree boosting method^22^ followed by an adaptive iterative search algorithm. The first module of the proposed ML approach quickly evaluates the importance of features (SNPs) to the BC risk prediction accuracy using an XGBoost model^22^. XGBoost is a fast and efficient implementation of the gradient tree boosting algorithm, which, in contrast to regression methods, considers non-linear interactions among features in a non-additive form. The first module provides an initial list of candidate BC-risk-predictive features. The second module then uses the candidate features in an adaptive iterative search to capture the interacting features that result in the best BC risk prediction accuracy on validation data (see Algorithm 1 in^21^ for details).

In particular, to combine the genetic variants with the demographic risk factors for BC (Group 1 and Group 2 features), we first identify the subset of interacting SNPs with the highest impact on BC risk using the ML approach described above. Then, two feature vectors are formed by concatenating the interacting SNPs separately with the Group 1 and Group 2 features. These feature vectors are then fed into the adaptive iterative search algorithm to identify the best combinations of interacting genetic, Group 1, and Group 2 features, which contribute most to BC risk prediction. Algorithm 1 describes this process.

It is worth noting that the adaptive iterative search algorithm ranks SNPs and demographic features from highest to lowest based on their BC-risk-predictive potential at each iteration. Therefore, several networks of interacting SNPs and demographic features are computed (one network per iteration). Features may overlap among networks. The iterative partitioning of data into training, validation and test sets places the BC cases (as well as controls) into different sets, which may track the possible heterogeneity among the BC cases and consequently lead to the identification of several networks of interacting genetic and demographic risk factors for BC.

To investigate the relative importance of each individual feature to the BC risk prediction performance on the KBCP test data, a feature vector is formed by leaving out each of the identified interacting features one at a time at each iteration; each time, the remaining features are used to train the XGBoost classifier from scratch. The importance of each feature is then computed by taking the average over all relative changes across iterations. This allows the importance of each individual feature to be measured relative to the system, which uses all the interacting features.

### Evaluation metrics

Since our dataset is partly imbalanced, we select the widely used precision-recall curve^27^ and mean average precision (mAP)^28^ as evaluation metrics to compare the performances of the different models in discriminating the BC cases and controls in the KBCP dataset. These are appropriate evaluation metrics for binary classifiers on imbalanced datasets^29^. The precision-recall curve illustrates the trade-off between precision and recall at different cut-off points^27^. The precision and recall are defined as^27^:

1 $${\rm{Precision}}=\frac{TP}{TP+FP}$$

2 $${\rm{Recall}}=\frac{TP}{TP+FN},$$

where TP = number of true positives, TN = number of true negatives, FP = number of false positives and FN = number of false negatives.

**Algorithm 1:**
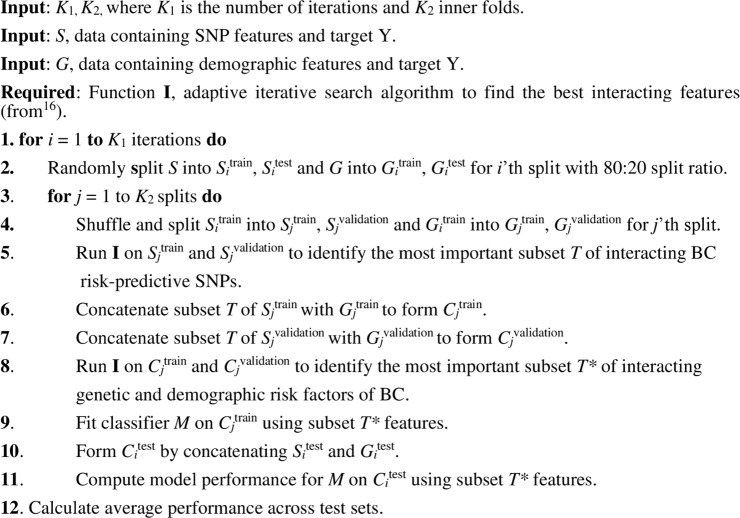
An iterative process to identify the best combination of factors of BC. The data does not overlap between training,validation and test subsets.

The average precision summarizes the precision-recall curve by computing the weighted mean of the precision at each cut-off point, using the increase in recall relative to the previous cut-off point as the weight^27^:

3 $${\rm{AP}}=\sum _{i}\,({{\rm{recall}}}_{i}-{{\rm{recall}}}_{i-1})\times {{\rm{precision}}}_{i}$$

where recall_*i*_ and precision_*i*_ are the precision and recall at the *i*-th threshold. The average precision is a single number between 0 (worst) and 1 (best) that indicates the average area under the precision-recall curve. In this study, the mAP is computed by averaging AP across multiple test sets.

### Implementation details

The proposed approach is implemented with XGBoost 0.6a2 and Python Scikit-Learn 0.18.2 using a Linux machine equipped with 56 CPUs and 240 GB memory provided by the IT Center for Science (CSC) Finland^30^. We pre-process the genotyped data by excluding the missing genotype values from the SNP data, resulting in 125,041 remaining SNPs. Then, the SNP data is encoded using an additive encoding scheme^31^ in which each SNP is represented by the minor allele count. The homozygous major and heterozygous and homozygous minor are encoded as 0, 1, and 2, respectively. We also transform the continuous variables of the Group 2 features by scaling each feature value to the range [0, 1] using min-max normalization^32^.

XGBoost is trained using logistic loss^33^ for binary classification, where the optimal hyperparameters are found at each iteration. To our knowledge, there are no recommendations in the literature on how to efficiently optimize the XGBoost hyperparameters when training on genotyped data. Therefore, we perform extensive XGBoost hyperparameter optimization; the hyperparameters include (I) the number of decision trees: the boosted trees are constructed sequentially by adding new trees (weak learners) to the model, where each new tree attempts to correct the errors made by the previous sequence of trees. The model often reaches a point where the addition of new trees does not improve the model performance. (II) The size of decision trees (tree depth): this is used to control over-fitting, as trees with higher depth generally learn too many details from the training samples. (III) The learning rate (shrinkage factor): this slows down the learning process in the gradient tree boosting model by reducing the impact of each individual tree on the estimates and leaving space for future trees to improve the model. (IV) The subsampling rate: this is the fraction of samples to be selected from the training data to create each tree. Random sampling without replacement is used to perform the selection. This simple technique (also called “stability selection”) adds variance to the ensembled estimation by allowing slightly different trees to be constructed from a random subset of the training data. In this study, we additionally optimized the XGBoost model for the following hyperparameters: (V) the minimum child weight: the minimum sum of instance weights in a tree node, (VI) gamma: the minimum loss reduction required to make a further partition of a leaf node of the tree, (VII) alpha: the L1 regularization term on weights, and VIII) the scale positive weight: this trains a class-weighted or cost-sensitive version of XGBoost for imbalanced classification and is typically set as the inverse of the class distribution in binary classification problems.

Using the training set (${S}_{i}^{train}$), a grid search over the triple of the number of decision trees, the size of the decision trees and the learning rate is first performed using 5-fold cross-validation (CV) at each iteration *i*; then, the subsampling rate, the minimum child weight, gamma, alpha and the scale positive weight hyperparameters are optimized in order according to the optimal hyperparameters found at each step. We followed the suggestions of^34,35^ for choosing 5-fold CV so that the size of the validation set (i.e., for the 5th fold, ${S}_{j}^{{\rm{validation}}}$ and ${G}_{j}^{{\rm{validation}}}$ in Algorithm 1) could be large enough to allow the most important interacting features to be identified.

### Baseline models for performance comparison

For comparison purposes, we use three individual systems based on the Group 1 features, the Group 2 features and the interacting BC-risk-predictive SNPs identified without considering the Group 1 and Group 2 features (denoted as ‘proposed SNPs’ in the results section). A PRS model is also derived from 82 published BC-risk associated SNPs extracted from^9,36,37^. Note that among the published BC risk-associated SNPs, 82 exist in our SNP discovery set. A list of the SNPs and their corresponding ORs are given in Supplementary Table S1. We also use the 82 published SNPs as feature vectors to train a BC risk prediction model. This system is denoted as ‘Literature SNPs’ in the results section. Additional baseline systems for comparison are constructed from the combinations of Literature SNPs + Group 1 features and Literature SNPs + Group 2 features.

The proposed approach as well as the baselines are evaluated in 10 repetitions of 5-fold stratified CV, keeping class frequencies balanced (*K*_1_ = 10, *K*_2_ = 5). This ensures that all classes are represented in all folds. The implementation source codes of the present study are freely available at https://github.com/hambeh/breast-cancer-risk-prediction.

### Genetic variant analysis

For genetic variant analysis, the overlapping genes within 5,000 bp upstream and downstream of each SNP are identified using Ensembl release 98^38^. To gain insight into the biological evidence of the SNPs that are found to interact with the demographic risk factors in the BC risk prediction task, we build a gene interaction map using the list of genes associated with those SNPs and the online tool esyN32 (www.esyN.org)^39^. esyN is an open source bioinformatics web tool for visualizing interaction data, in which nodes represent biological entities (e.g., genes, proteins, molecules) and the interactions between them are represented by edges connecting the nodes.

## Results

### Optimizing the XGBoost hyperparameters

The XGBoost model is first optimized in the context of BC risk prediction using genotyped data. The combination of all hyperparameters optimized at each iteration of Algorithm 1 is summarized in Table 3 and illustrated in Supplementary Fig. S1. We can see that fewer boosted trees with smaller values of tree depth were found to be optimal. As suggested by^40^, overfitting often occurs when increasing the number of boosted trees with more depth. The optimal tree depth was found to be 2 in the majority of the iterations except the fifth iteration. We also found that the optimal value of the learning rate is 0.01 for all iterations. Smaller values of the subsuming rate, such as 40% to 60%, resulted in the best prediction performance on the validation data, which is in line with the findings of Friedman^40^. The optimal values of the minimum child weight, gamma and alpha, however, varies among iterations. Interestingly, the proposed approach achieves a higher prediction performance on the validation data when the scale positive weight is 1 than when it is 0.56, that is, the inverse of the class distribution ($=\frac{\#{\rm{Controls}}}{\#{\rm{Cases}}}$). In practice, tuning the scale positive weight hyperparameter works fairly well when the dataset is extremely unbalanced (for example, when more than 95 % of the training instances are labelled with the majority class)^41^, which is not the case in this study.

**Table 3:** Combination of the XGBoost hyperparameter settings and their optimal values found at each iteration of Algorithm 1.

Iteration	No. of decision trees*	Tree depth*	Learning rate*	Sub-sampling rate*	Min.child weight	Gamma	Alpha	Scale positive weight
Search range	[50, 100, 150, 200, 250, 300]	[2, 4, 6, 8]	[0.001, 0.01,0.1]	[0.1, 0.2, 0.3, 0.4, 0.5, 0.6, 0.7, 0.8, 0.9, 1]	[0, 0.1, 0.2, 0.5, 0.6, 0.7, 0.8, 0.9, 1, 2, 3, 4, 5]	[0, 0.1, 0,2, 0.3, 0.4, 0.5, 0.6, 0.7, 0.8, 0.9, 1, 4, 9]	[1e-5, 1e-2, 0.1, 1, 2, 5, 100]	[0.56, 1]
1	200	2	0.01	0.4	0	0	2	1
2	150	2	0.01	0.4	2	9	1e-05	1
3	150	2	0.01	1	2	0.4	0.01	1
4	100	2	0.01	0.4	0	0	2	1
5	150	4	0.01	0.6	1	0.1	1e-05	1
6	150	2	0.01	0.3	2	4	0.01	1
7	150	2	0.01	0.6	4	9	5	1
8	150	2	0.01	0.2	3	0.4	1e-05	1
9	200	2	0.01	0.5	3	4	1e-05	1
10	100	2	0.01	0.1	0.7	0.1	1e-05	1

*Optimal values obtained from our previous study^21^.

### Effect of the number of BC-risk-predictive SNPs on the risk prediction

**Figure 2:**
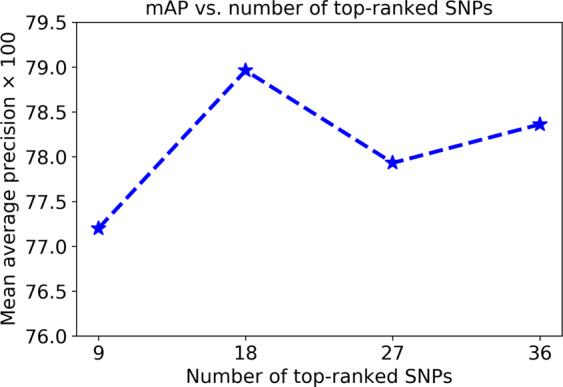
Prediction performance in terms of the mAP via the increase of the number of top-ranked SNPs when they interact with the Group 1 features. The results are reported on the validation data.

To identify the best combinations of the interacting genetic, Group 1 and Group 2 features, we need to specify the optimal number of SNPs concatenated to the Group 1 and Group 2 features to form the feature vectors ${C}_{j}^{{\rm{train}}}$ and ${C}_{j}^{{\rm{validation}}}$ in Algorithm 1. Figure 2 displays the BC risk prediction accuracy in terms of mAP via the increase of the number of top-ranked SNPs on the validation set. As shown, the best accuracy is achieved when 18 top-ranked SNPs are concatenated to the Group 1 features. Based on this finding, the 18 top-ranked SNPs at each iteration are used to find the best combinations of genetic and demographic risk factors in the subsequent experiments.

### Effect of the training/test split ratio and number of iterations on the prediction performance

**Figure 3:**
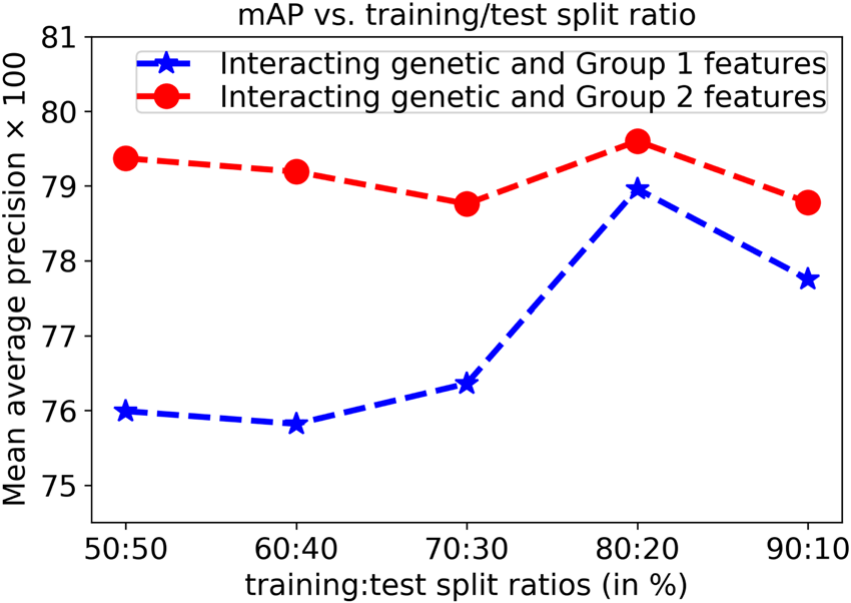
Prediction performance in terms of the mAP as a function of the training/test set split ratio using the validation data.

Figure 3 further demonstrates the prediction performance in terms of mAP as a function of the training/test set split ratio on the validation set. The mAPs are reported for each ratio using 10 iterations of Algorithm 1 (*K*_1_ = 10). The best mAPs, of 78.96 and 79.60, are achieved for the interacting genetic and Group 1 and 2 features, respectively, using a ratio of 80:20 to split the data (80% for the training set and 20% for the test set). Increasing the training size up to 80% gradually increases the mAP; however, at a 90:10 ratio, the mAP decreases to 77.75 and 78.78 for the interacting genetic and Group 1 and 2 features, respectively, which indicates that the test set size (10% of the data) may not be a reasonable representative of the problem for evaluation.

**Figure 4:**
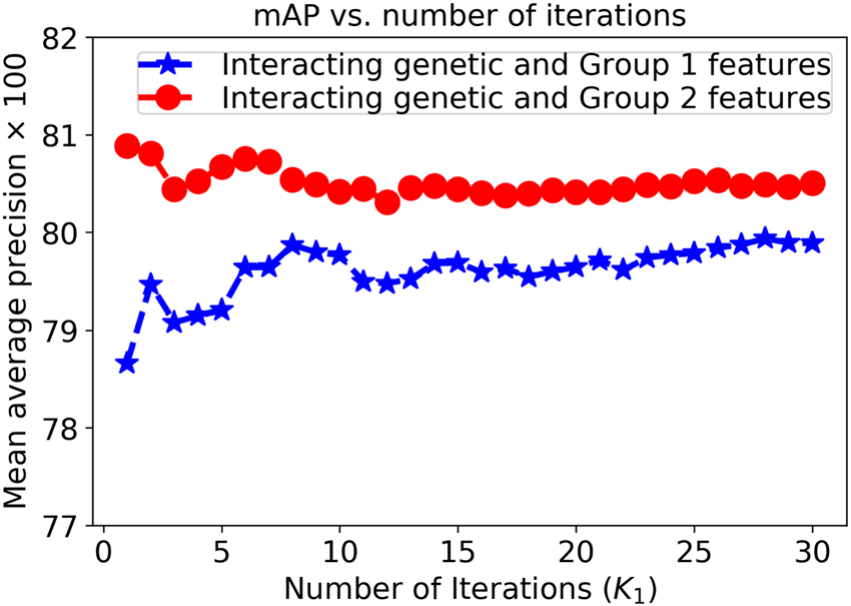
Prediction performance in terms of the mAP as a function of the number of iterations (*K*_1_ in Algorithm 1) using the validation data.

Similarly, Fig. 4 shows the prediction performance in terms of the mAP as a function of the number of iterations (*K*_1_ in Algorithm 1) on the validation set. The data are partitioned randomly into training and test sets using an 80:20 split ratio at each iteration, and the mAP is the average of the prediction performance across all validation sets. The results in Fig. 4 show that after 10 iterations of Algorithm 1, the mAP reaches a plateau, and increasing the number of iterations no longer affects the prediction performance of either system.

Based on these findings, we select an 80:20 training/test split ratio at each iteration and fix *K*_1_ = 10 for the rest of the experiments.

### Breast cancer risk prediction using interacting genetic, Group 1 and Group 2 features

**Figure 5:**
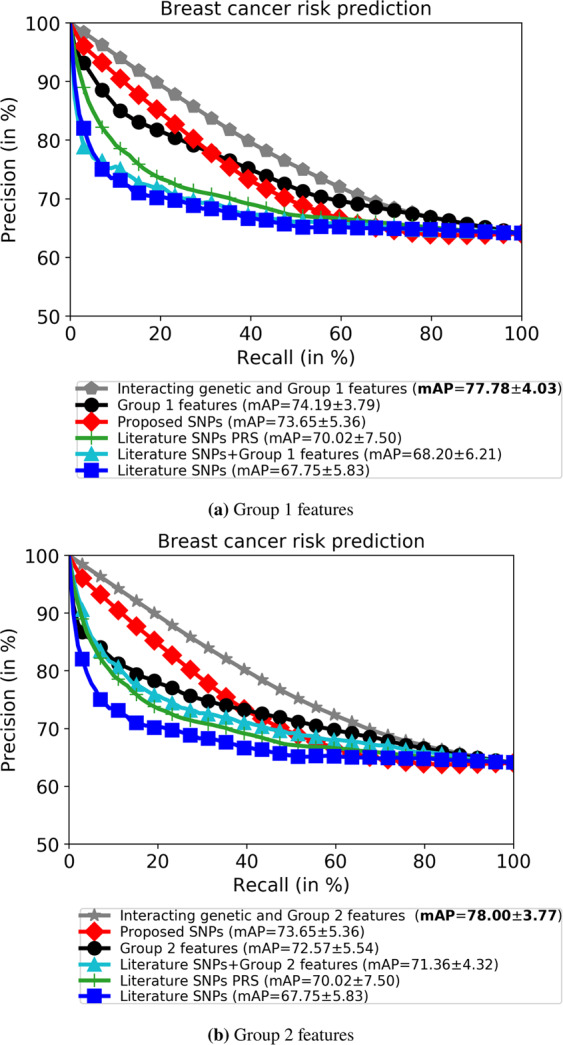
Combining the Group 1 (**a**) and Group 2 (**b**) demographic features with the genotyped data outperforms all other single systems in BC risk prediction on the KBCP test data. Literature SNPs denotes the system trained on 82 known BC-risk-associated SNPs, and the PRS model is derived from those 82 SNPs. The number after ± denotes the standard deviation. The high standard deviations are due to multiple subset selections.

Next, we illustrate the precision-recall curve comparison between the proposed BC risk prediction method and five baseline systems in terms of discriminating the BC cases and controls on the KBCP test data. The results demonstrated in Fig. 5(a) indicate that the system based on the interacting genetic and Group 1 features achieves a mAP of 77.78, which is higher than the mAPs of 74.19 and 73.65 obtained by the systems that use only the Group 1 features and the proposed SNPs (855 SNPs), respectively. The PRS model based on the 82 literature SNPs achieves a mAP of 70.02, outperforming the system based on the 82 literature SNPs with a mAP of 67.75. Interestingly, our proposed system (first row of Fig. 5(a)), with a mAP of 77.78, outperforms the system based on the literature SNPs + Group 1 features, with a mAP of 68.20; this highlights the importance of allowing the networks of interacting SNPs to interplay with the Group 1 features to find the best combinations of SNPs and Group 1 features in the BC risk prediction task.

Similarly, Fig. 5(b) shows that the system based on the interacting genetic and Group 2 features achieves a mAP of 78.00, and it outperforms the systems that use only the Group 2 features, with a mAP of 72.57, and the proposed SNPs (855 SNPs)a with a mAP of 73.65. By comparing Fig. 5(a,b), one can see that the system based on the Group 1 features achieves a better mAP, of 74.19, compared to the Group 2 features, with a mAP of 72.57. Similar to the above, our proposed system (first row of Fig. 5(b)), with a mAP of 78.00, outperforms the system based on the literature SNPs + Group 2 features, with a mAP of 71.36.

The above results show that a combination of interacting genetic and demographic risk factors yields the highest BC risk prediction accuracy, highlighting the interplay between genetic and demographic risk factors in a multifactorial disease such as BC. Notably, the BC risk prediction model works particularly well when the interaction between the SNPs and demographic risk factors is investigated among different sets of interacting SNPs, compared to combining only the known BC-risk-associated SNPs (82 SNPs) with the demographic risk factors.

To evaluate the robustness and overfitting of the proposed ML approach, Table 4 gives the BC risk prediction accuracy in terms of mAP on the training, validation and test sets of the KBCP dataset. In both systems, the prediction accuracies of the training set and the validation/test sets are comparable. The results indicate that the proposed approach does not overfit the training data and performs favourably on both the validation and test sets.

**Table 4:** BC risk prediction accuracy in terms of mAP of the training, validation and test sets of the KBCP dataset. The results indicate the robustness of the proposed method in discriminating BC cases and controls.

Proposed approach	Training set	Validation set	Test set
Interacting genetic and Group 1 features	80.25 ± 2.66	78.96 ± 2.78	77.78 ± 4.03
Interacting genetic and Group 2 features	80.19 ± 2.12	79.60 ± 2.28	78.00 ± 3.77

### Effect of individual features on the prediction performance

**Figure 6:**
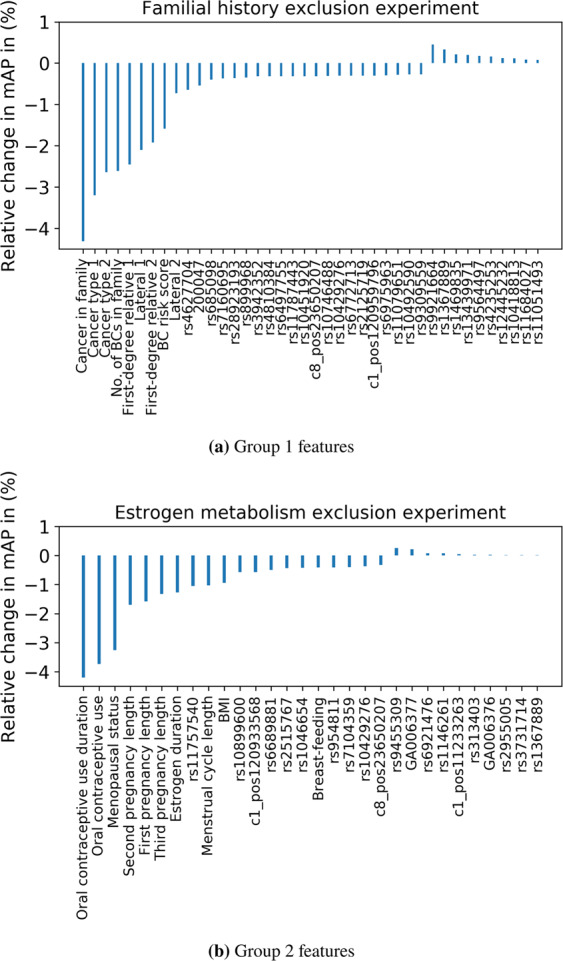
Relative change in mAP as each feature is left out. A negative relative change indicates a reduction in the BC risk prediction performance. (**a**) relative change of the mAP in the Group 1 experiment. (**b**) relative change of the mAP in the Group 2 experiment.

We now investigate the relative importance of each individual feature to the BC risk prediction performance on the KBCP test data. Recall that when using all the identified interacting features, the mAP is 77.78 and 78.00 for systems based on the interacting genetic and Group 1 and 2 features, respectively. Figure 6 reveals that leaving out any of the demographic features (either Group 1 or Group 2 features) from the final feature vector decreases the mAP in both Group 1 and Group 2 experiments, indicating the importance of these features in discriminating the BC cases and controls. The relative change in the mAP is smaller for the individual SNPs compared to the individual Group 1 and Group 2 features, indicating the importance of considering groups of SNPs and their interactions in the BC risk prediction task. Figure 6(a) shows that among the Group 1 features, cancer in the family and cancer type are individually the most important features, with 4.5% and 3% negative relative change in mAP, respectively, while the BC risk score and lateral 2 are less important features, with 2% and 1.5% negative relative change in mAP, respectively. Note that negative relative change indicates reduction in BC risk prediction performance. Similarly, Fig. 6(b) shows that among the Group 2 features, oral contraceptive use and menopausal status are individually the most important, with 4% and 3% negative relative change in mAP, respectively, while BMI and breast-feeding are less important, with 1% and 0.5% negative relative change in mAP, respectively. Interestingly, the top-ranked SNP in Fig. 6(b), rs11757540, is linked to the *ESR1* gene, which is one of the important oestrogen metabolism-related genes in BC development^42^.

The individual assessment of features by statistical tests may partly explain the order of the demographic features shown in Fig. 6. Among the Group 1 features, cancer in the family, cancer type 1, first-degree relative 1, no. of BC cases in the family and the BC risk score are found to be significantly different between the cases and controls (p-value <= 0.5, last column of Table 1). Most of these features are marked as the most important features in Fig. 6(a). Among the Group 2 features, there is no significant difference in menstrual cycle length and BMI between the BC cases and controls (p-value > 0.5, last column of Table 2). These features are also marked as less important among the Group 2 features in Fig. 6(b). Similarly, the top Group 2 features in Fig. 6(b), such as oral contraceptive use, menopausal status and second pregnancy length, differ significantly between the BC cases and controls, as shown in Table 2.

**Figure 7:**
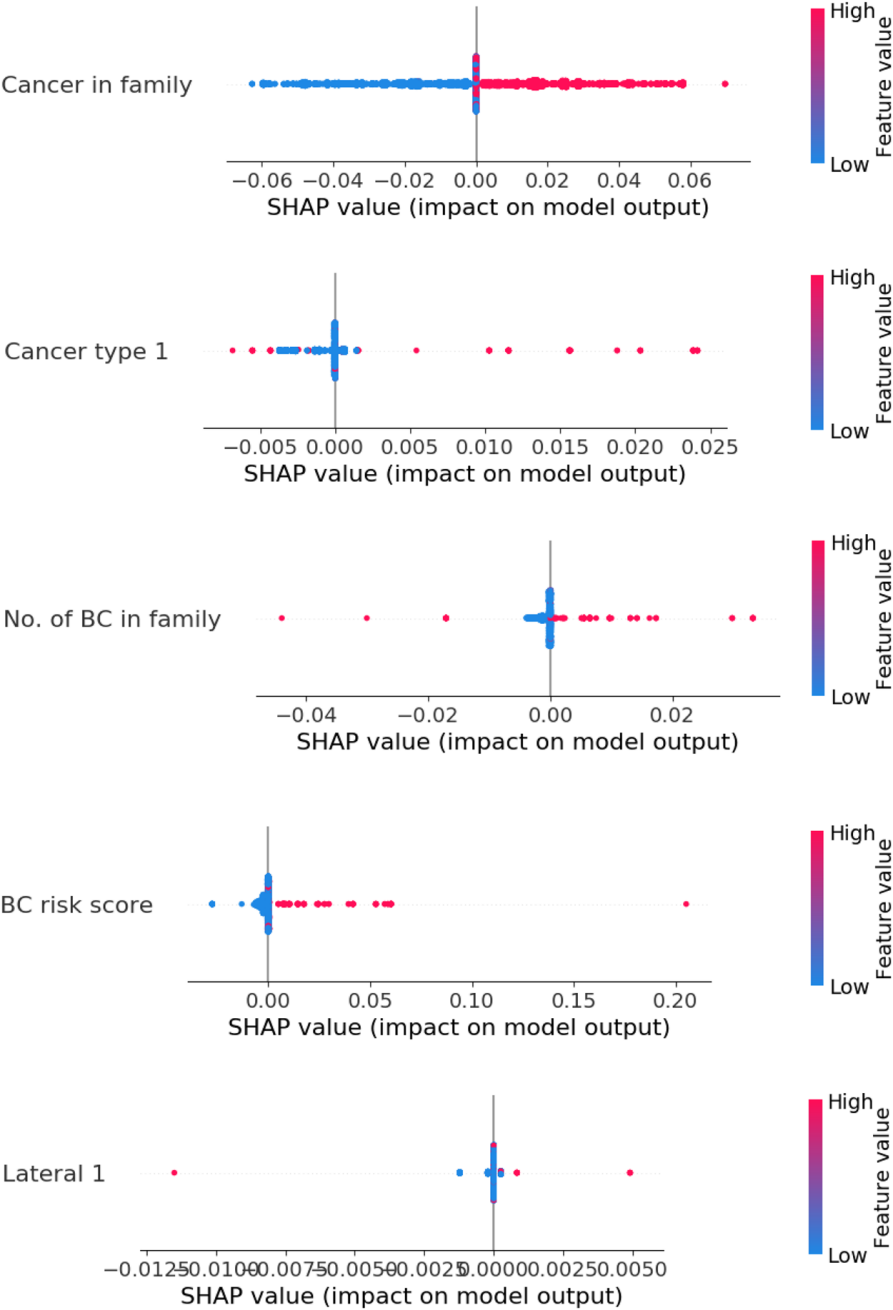
An overview of the impact of each feature value in Group 1 on the model output using the SHAP value. The colour represents the feature value (red is high, blue is low). Each point indicates the SHAP value of a specific feature for each KBCP test data point. The SHAP algorithm^44^ uses a linear model and all possible combinations of features with and without a specific feature to find the importance of a feature in a prediction task in terms of the log-odds change in the prediction. The farther the dot is to the right on the x-axis, the greater the influence on the model to predict ‘case’ for that particular sample.

**Figure 8:**
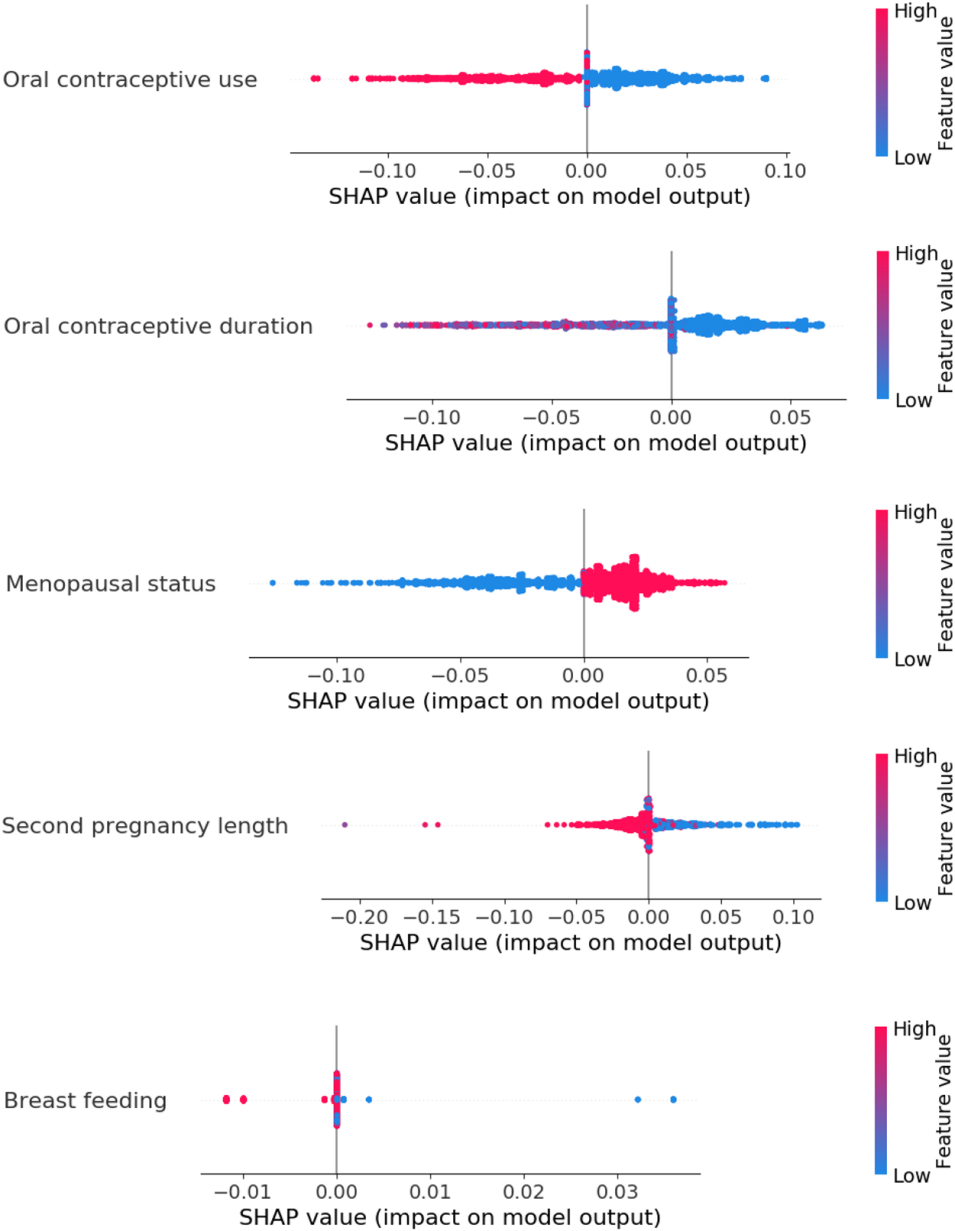
An overview of the impact of each feature value in Group 2 on the model output using the SHAP value.

Figures 7 and 8 further illustrate the distribution of the impacts of different values of the selected Group 1 and Group 2 features on the model output using their SHAP values^43^. In the figures, an increasing BC risk indicates that the value of a feature shifts the model output to the right on the x-axis, towards predicting a case, while a decreasing BC risk indicates that the value shifts the model output to the left, towards predicting a control. Figure 7 reveals that among Group 1 features, having cancer in the family (red dots), breast as cancer type (red dots), more family members with BC (red dots) and a higher BC risk score (red dots) increase the BC risk, as one may expect. Among the Group 2 features in Fig. 8, using oral contraceptives (red dots) and breast-feeding (red dots) decrease the BC risk, while a shorter pregnancy length (blue dots) and menopause (red dots) increase the BC risk. The results obtained seem reasonable. For example, it is known that women who have close relatives with BC have a higher risk of developing BC^23^ and that a reduced risk of BC is associated with full-term pregnancies lasting 34 weeks or longer^44^. The use of oral contraceptives has been shown to slightly increase BC risk^45^, but our results show the opposite. From Table 2, most of the cases (67%) in the KBCP dataset did not use oral contraceptives, and therefore, the model learnt this property from the data. The development of contraceptives in the time since the women recruited for the KBCP were using contraceptives may partly explain this discrepancy. The effect of menopause can also be attributed to age, as the risk of developing BC increases as a woman ages. See the Supplementary Figs. S2 and S3 for a full analysis of each feature’s value impact for the Group 1 and Group 2 features.

Note that while not all the feature contributions to the model output necessarily contribute causally to the BC risk, we still observe that for most of the features, the obtained results agreed well with the established impact of those features on the BC risk; this indicates that the proposed approach finds true patterns in the input data and trains the model for BC risk prediction. Next, we perform a gene interaction analysis of the identified SNPs found to interact with the demographic risk factors to study whether those SNPs involve any biological pathways of BC.

### Analysis of the genes associated with the SNPs that were found to interact with the demographic risk factors

A total of 139 and 145 SNPs were found to interact with the Group 1 and Group 2 features, respectively, when training the BC risk prediction system. Interaction means that the features are selected together during the iterative process of feature selection. A gene interaction map was built from the genes associated with those SNPs. A total of 86 and 90 genes were associated with the SNPs that interact with the Group 1 and the Group 2 features, respectively. The details of the SNPs and their associated genes are given in Supplementary Tables S2 and S3 for the Group 1 and Group 2 features, respectively.

**Figure 9:**
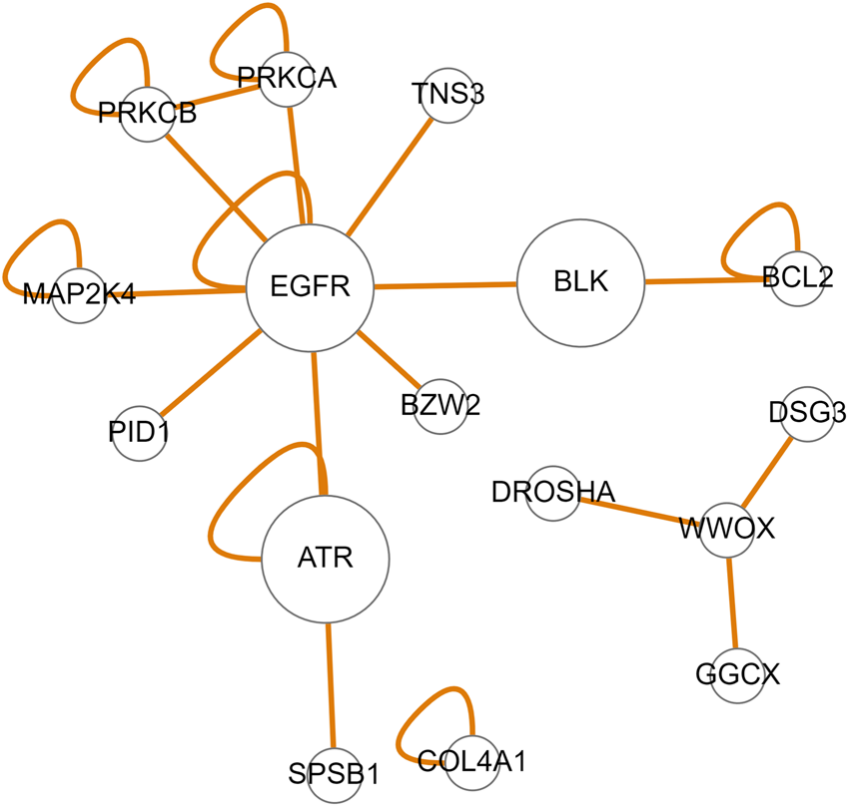
Gene interaction map of the genes associated with the SNPs that were found to interact with the group 1 features in the KBCP BC risk prediction task.

The gene interaction map of the SNPs that were found to interact with the Group 1 features reveals three individual cancer-related networks, among which the *EGFR*-*BLK*-*ATR*- and *WWOX*-linked networks are noticeable (Fig. 9). Activation of the *EGFR* signalling pathway in cancer cells has been linked with tumour growth, increased cell proliferation, angiogenesis, metastasis, and inhibition of apoptosis^46^. In BC, high expression of *EGFR* was related to *BRCA1* or *BRCA2* mutations^47^, and *EGFR* polymorphism pointed to a possible inheritance of cancer risk associated with the *EGFR* gene^48^. It is also suggested that there are fundamental changes in *EGFR* signalling that take place during primary tumour invasion, dissemination and the ultimate metastasis of BC cells^49^. *ATR* encodes a serine/threonine kinase that has a role as a DNA damage sensor and in activating cell cycle checkpoint signalling upon DNA stress. It is also a known BC susceptibility gene shown to phosphorylate several tumour suppressors such as *BRCA1*, *CHEK1* and *TP53*^50,51^. Note that mutations in known BC susceptibility genes, such as *BRCA1*, *BRCA2* and *TP53*, are suggested to be responsible for approximately 25% of the familial component of BC risk^52^. The *BLK* oncogene is a member of the SRC family of protein tyrosine kinases typically involved in cell proliferation and differentiation, and abnormal expression of it was found in several malignancies, including breast and colon cancers^53^. *WWOX*, a tumour suppressor gene, is involved in several biological signalling pathways, including regulating cell apoptosis and differentiation. Deletions within the *WWOX* coding sequence are observed in up to 80% of BC cases^54^. Copy number variations of the *WWOX* gene were found to be a susceptibility factor in familial BC^55^.

**Figure 10:**
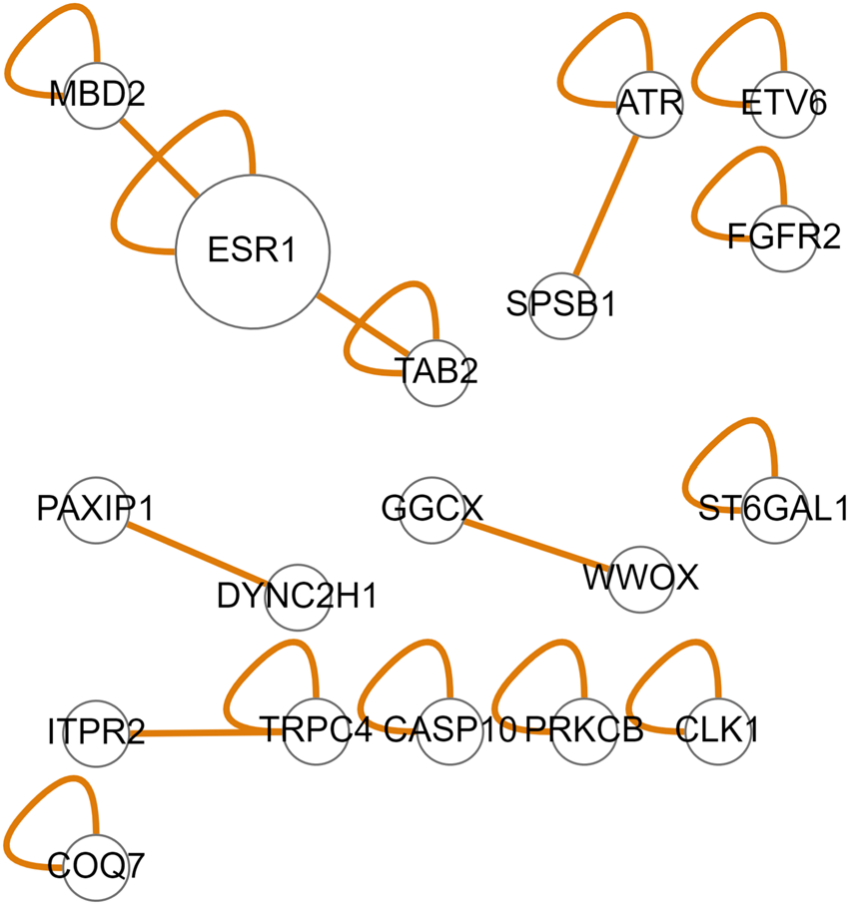
Gene interaction map of the genes associated with the SNPs that were found to interact with the group 2 features in the KBCP BC risk prediction task.

Interestingly, the gene interaction map of the SNPs that were found to interact with the Group 2 features points to the prominent oestrogen-related *ESR1*-linked network (Fig. 10). Figure 10 additionally points to the *FGFR2* gene, which is one of the most important genetic susceptibility loci in BC^56^. A locus within the second intron of the *FGFR2* gene is consistently identified as the genetic locus most strongly associated with oestrogen receptor (ER)-positive (ER+) BC risk by independent GWAS analyses^57^. The most significant-risk SNPs^58,59^ act to reduce *FGFR2* gene expression and enhance the oestrogen response^60^. Increased *FGFR2* stimulation repressed oestrogen signalling in ER+ BC cell lines. However, the underlying molecular mechanism remains unclear. *FGFR2* also transactivates *HER2* via c-SRC, leading to resistance to *HER2*-targeted therapies.

## Discussion

New discoveries in recent years have identified a number of risk factors contributing to BC risk, ranging from the genetic variants identified in GWASs to BC risk factors related to familial history and oestrogen metabolism. Merging genetic and non-genetic risk factors could enable the development of risk-adapted screening programs, which can, in turn, categorize individuals based on their risk of developing cancer and then send those with a high risk of developing cancer for more precise screening, e.g., by performing mammography, MRI and/or tumour segmentation. This could potentially improve the performance of BC screening and lead to an efficient allocation of clinical resources^61,62^.

Although risk factors have been found to be individually important for BC risk evaluation, few studies have considered combining several risk factors for cancer risk prediction. One major challenge in BC risk prediction is to develop a model that incorporates all known and newly found risk factors while considering the interactions among them. Through the breast cancer association consortium (BCAC) data^63^, it is now possible to study the effect of multiple SNPs using increasingly large GWAS- and PRS-derived models. GWASs have successfully identified more than 200 SNPs significantly associated with BC risk in case-control studies^9,36,37^. The identified SNPs explain the genetic component of the BC risk to some extent. In a recent attempt, a network-based GWAS approach was used to identify genetic determinants of BC prognosis among ~7.3 million imputed genetic variants using a one-by-one association analysis^64^. Summary statistics of the individual variants were then aggregated into gene-level *p*-values to identify gene modules associated with survival in ER+ and ER− disease. However, this has limitations, as single SNPs have a small effect size, and SNPs are identified independently, without considering the possible interactions and correlations among them. With the help of ML methodologies, we propose that it is now possible to consider the joint effects of risk-associated SNPs. We have already shown that single SNPs are not as important as networks of interacting SNPs identified by an ML approach in performing the BC risk prediction task^21^. However, as BC is a multi-factorial disease, genetic variants cannot alone provide a comprehensive view of the components of the disease risk^65^. This supports the idea that the explanatory power of genetic variants could be enhanced by combining them with other sources of individual variation, such as demographic risk factors, as shown in this study.

Using regression methods, previous studies have shown that established genetic and environmental risk factors are likely to combine multiplicatively in their associations with BC risk; however, such methods often had limited capability in detecting interactions^66–68^. Regression methods often assume that risk factors are independent of each other and interact in a linear way to promote BC development. We have already shown in^21^ that in a BC risk prediction task, capturing the complex interactions among SNPs with an iterative search and a gradient-boosting method outperforms the classical linear penalized logistic regression method and a PRS model derived from established BC-risk-associated SNPs. In particular, our study differs from similar studies^6,15^ in incorporating a non-linear feature selection algorithm and allowing the ML model to find the best networks of interacting genetic and demographic risk factors that contribute most to BC risk prediction. We showed that such a system considerably outperforms a system that combines known BC-risk-associated SNPs and demographic risk factors (Group 1 and Group 2 features) without considering the interactions among them. Furthermore, a gene interaction map created from the genes associated with the SNPs that were interacting with the demographic risk factors pointed to important biological pathways involved in BC development, such as apoptosis, angiogenesis and oestrogen-related entities, which are established biological networks in BC development. Note that the identified genes and pathways might point to biological networks with no known effect on BC development.

Strategies based on predictive genomics and cancer hallmarks for cancer biomarker identification have also been published to predict cancer risk and patient outcomes^69–72^. These strategies often measure alterations in pre-defined cancer susceptibility genes. Apart from successfully generating robust cancer prognostic and diagnostic gene signatures, these strategies are often limited to a set of pre-selected candidate genes. The proposed approach in this study, which is free from pre-selection of risk factors (e.g., SNPs), can be integrated with hallmark-based strategies to further enhance predictions of cancer risk and search for optimal interactions. Indeed, the present study can also be extended to other multifactorial diseases.

In this study, demographic risk factors were also found to be individually important in our BC risk prediction model, although their importance scores were not equal. Similar to the genetic variants, combinations of demographic risk factors yielded a higher risk prediction accuracy than the individual demographic risk factors. Interestingly, we observed a mAP of 78.36 when the Group 1 and Group 2 features were combined and interacted with the genotyped data (15 top-ranked SNPs in each iteration), which is higher than the previously best system based on interacting genetic and Group 2 features, with a mAP of 78.00.

A limitation of the present study is the small and partly imbalanced amount of data used to train the BC risk prediction model, which might cause overfitting and impact prediction performance. The small amount of data is due to the difficulty of collecting a dataset that contains different types of risk factors. In this study, we addressed the data imbalance issue at three levels: i) data splitting, ii) model optimization, and iii) model evaluation. First, due to the class imbalance, fold assignments in CV were performed in a stratified manner, ensuring that all classes were represented in all folds. Then, appropriate parameter optimization was performed to ensure that the model was well generalized and predictive. Specifically, we experimented with the effect of weighting with inverse class frequencies and fine-tuned the minimum sum of instance weights in a tree node to compensate for the class imbalance during training. Finally, the proposed approach as well as the baselines were all evaluated in 10 repetitions of 5-fold CV using the precision-recall curve and mAP for performance comparison. Note that the evaluation strategy not only reduced the risk of overfitting (Table 4) but also placed subjects in non-overlapping training, validation and test sets, which allowed multiple networks of interacting genetic and demographic risk factors (10(iterations) × 5(CV) = 50 networks) to be identified, corresponding to each subset of data and possible heterogeneity among BC cases.

Finally, we plan to validate our results using an extended dataset obtained from the Biobank of Eastern Finland^73^. Using the extended dataset, we can also increase our minimal class sample size (controls).

## Conclusion

In this study, we proposed an ML approach to efficiently combine genetic variants with BC risk factors related to both familial history and oestrogen metabolism and to search for optimal interactions among them. The proposed approach considerably increased the BC risk prediction accuracy compared to systems based solely on genetic variants or demographic risk factors for BC. To summarize, the main contributions of the present study are as follows: i) identifying the networks of interacting genetic and demographic risk factors for BC that contribute most to predicting the BC risk, ii) proposing an efficient ML framework to combine different risk factors for a multifactorial disease such as BC in a high-dimensional and partly small-sample-size problem, iii) capturing non-linear interactions among the risk factors and modelling BC risk in a non-additive form, and iv) constructing a gene-gene interaction map of the SNPs that were found to interact with the demographic risk factors and finding their relevance to important biological entities for BC, such as apoptosis, angiogenesis and oestrogen-related metabolism. In future, our results will help to create more effective ways to identify people at risk for BC, to whom screening methods should be directed. Our model is also adaptable to all other multifactorial disease entities.

## Supplementary information

Supplementary Information.

## Data Availability

The datasets generated and/or analysed during the current study are available from the corresponding author on reasonable request.
