# Supplementary material for: Predicting breast cancer risk using interacting genetic and demographic factors and machine learning
Source: Sci Rep. 2020 Jul 6;10:11044. doi: 10.1038/s41598-020-66907-9 (PMC7338351; doi:10.1038/s41598-020-66907-9)
Supplement: Supplementary file 1 — Supplementary Information. [file 41598_2020_66907_MOESM1_ESM.pdf]

Predicting breast cancer risk using interacting genetic and demographic factors and machine learning

Hamid Behravan[*1], Jaana M. Hartikainen[1], Maria Tengström[3,4], Veli–Matti Kosma[1,2,†] & Arto Mannermaa[1,2,†]

[1] Institute of Clinical Medicine, Pathology and Forensic Medicine, and Translational Cancer Research Area, University of Eastern Finland, P.O. Box 1627, FI-70211, Kuopio, Finland.
[2] Biobank of Eastern Finland, Kuopio University Hospital, Kuopio, Finland.
[3] Institute of Clinical Medicine, Oncology, University of Eastern Finland, P.O. Box 1627, FI-70211, Kuopio, Finland.
[4] Cancer Center, Kuopio University Hospital, Kuopio, P.O. Box 100, FI-70029, Kuopio, Finland
[†] These authors contributed equally.
* Corresponding author: hamid.behravan@uef.fi

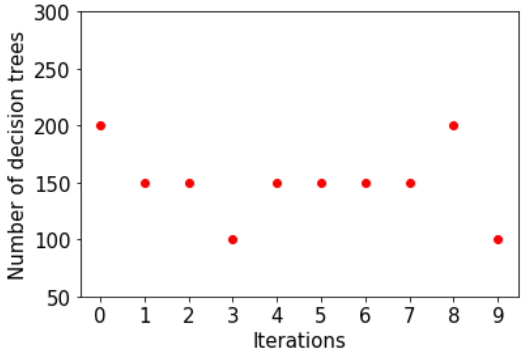

a) Number of decision trees

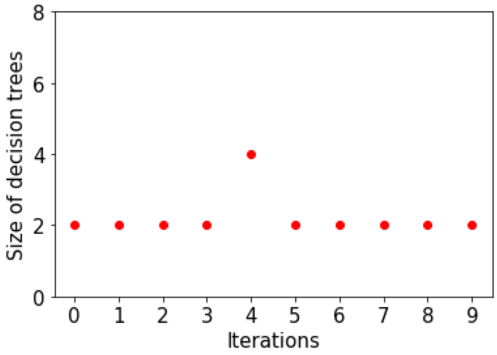

b) Size of decision trees

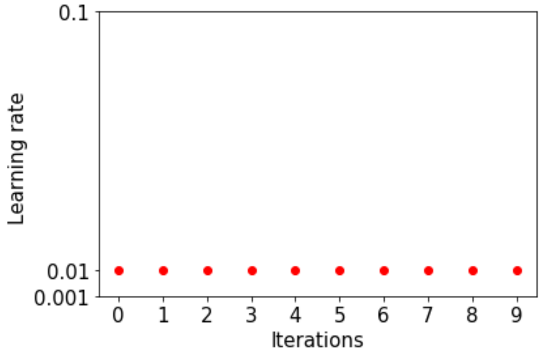

c) learning rate

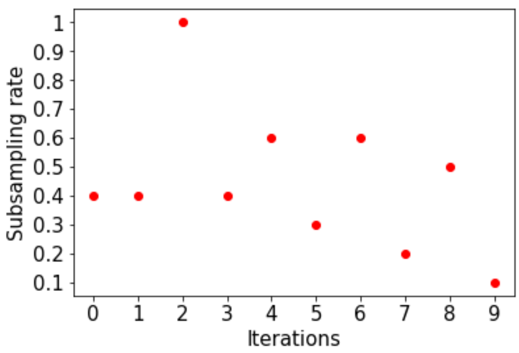

d) subsampling rate

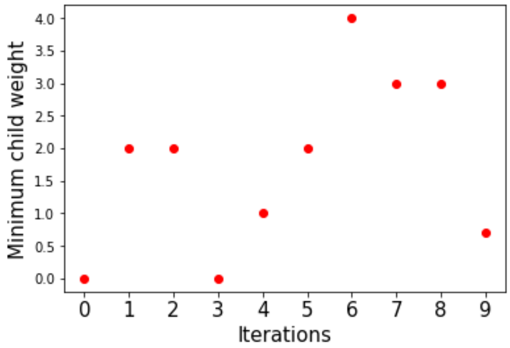

e) Minimum child weight

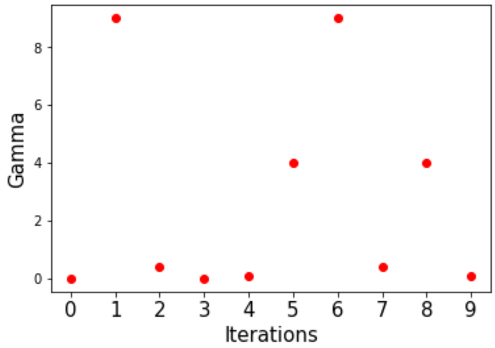

f) Gamma

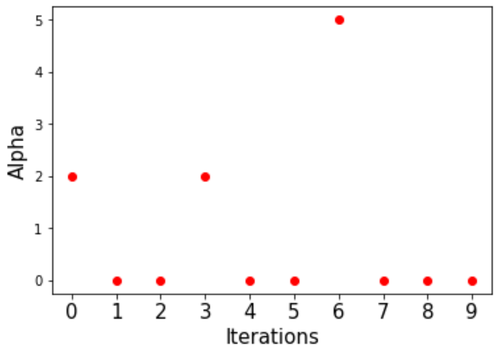

g) Alpha

Figure S1: The optimal values of the XGBoost hyperparameters obtained at each iteration.

Table S1: List of 82 published BC-associated SNPs and their corresponding odd ratio (OR) value from [9,37,38].
*OR value and effect allele were obtained from GWAS Central (http://www.gwascentral.org/).

**SNP name**	**Odd ratio**	**Effect allele**
rs616488*	1.1	A
rs4245739	1.03	C
rs12710696	1.04	A
rs4849887*	1.1	G
rs1550623*	1.06	A
rs6762644	1.07	G
rs1053338	1.08	G
rs7726159	1.04	A
rs2736108	1.07	G
rs889312	1.12	C
rs10472076	1.05	G
rs1353747*	1.09	A
rs1432679	1.07	G
rs11242675*	1.06	A
rs17529111	1.06	G
rs12662670	1.17	C
rs2046210	1.08	A
rs9693444	1.07	A
rs11780156	1.07	A
rs1011970	1.06	A
rs10759243	1.05	A
rs865686*	1.12	A
rs7072776	1.07	A
rs11814448	1.27	C
rs704010	1.08	A
rs7904519	1.05	G
rs11199914*	1.05	G
rs2981579	1.27	A
rs3817198	1.07	G
rs554219	1.26	G
rs11820646*	1.05	G
rs12422552	1.04	C
rs10771399*	1.16	A
rs17356907*	1.1	A
rs1292011*	1.09	A
rs11571833	1.27	T
rs2588809	1.08	A
rs999737*	1.09	G
rs941764	1.07	G
rs3803662	1.24	A
rs17817449*	1.09	A
rs11075995	1.04	A
rs13329835	1.08	G
rs6504950*	1.06	G
rs527616*	1.05	G
rs1436904*	1.04	A
rs2363956	1.03	A
rs4808801*	1.08	A
rs2823093*	1.09	G
rs132390	1.14	G
rs6001930	1.12	G
rs637868	1.04	G
rs17426269	1.05	G
rs16886165	1.23	C
rs11949391	0.9	G
rs10022462	1.04	G
rs17156577	1.05	G
rs17350191	1.07	G
rs2380205	1.06	G
rs3757322	1.08	C
rs2747652	1.06	G
rs10816625	1.11	G
rs13294895	1.06	G
rs4577244	1.08	G
rs7297051	1.05	G
rs6562760	1.05	G
rs1219648	1.22	G
rs2981575	1.28	G
rs2981582	1.18	G
rs2420946	1.19	G
rs1641535	1.17	G
rs231775	1.04	G
rs4919682	1.04	G
rs1695	1.16	G
rs1800067	1.08	G
rs1056836	1.08	G
rs4986938	0.94	G
rs1801516	1.18	G
rs1801270	2.08	C
rs2287499	1.08	G
rs570613	1	G
rs1801320	1.04	G

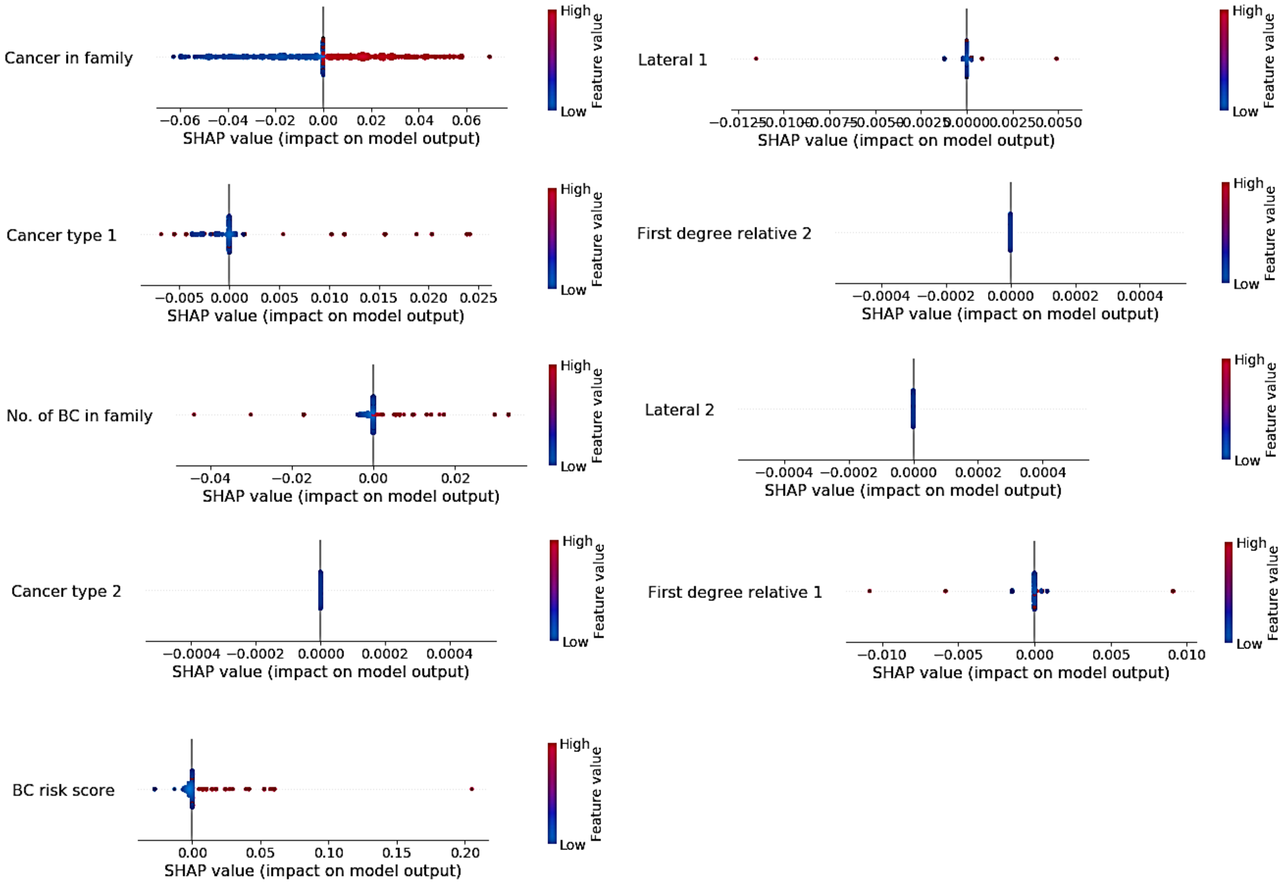

Figure S2: Full feature's value contribution analysis of the Group 1 features using SHAP value [44].

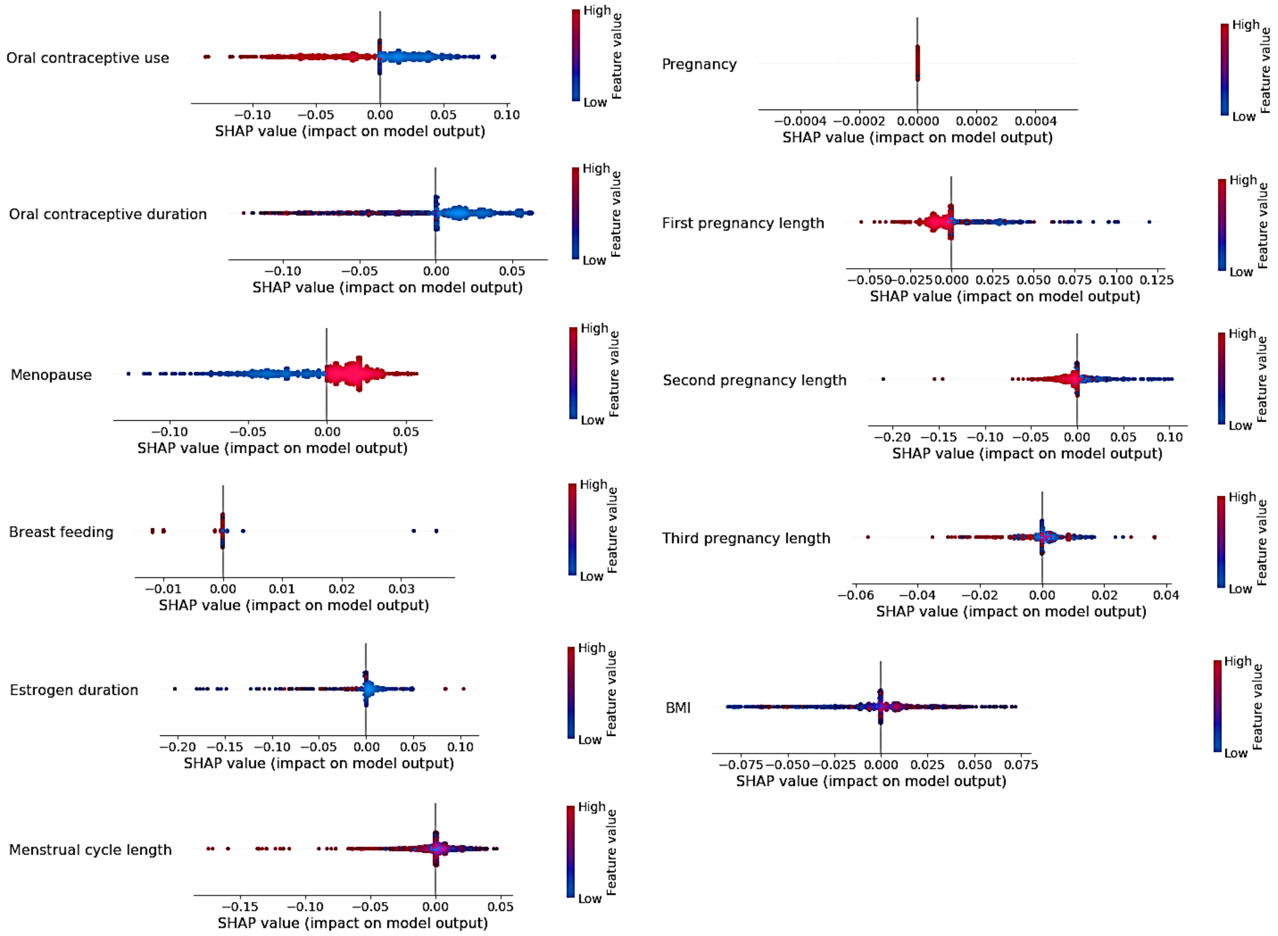

Figure S3: Full feature’s value contribution analysis of the Group 2 features using SHAP value.

Table S2: SNPs found interacting with the Group 1 features in the BC risk prediction task and their associated genes.

**Variant**	**Chromosome**	**Position start (bp)**	**Associated gene name**
rs2043885	2	124098762	CNTNAP5
rs1466379	18	31476502	DSG3
rs12764105	10	54718524	PCDH15
rs899968	18	63155031	BCL2
rs7129973	11	89182402	TYR
rs1328710	6	71892861	RIMS1
rs2275060	10	70530022	PALD1
rs4937216	11	126898194	KIRREL3
rs2955005	8	81706019	ZFAND1
rs13439971	9	94151482	AL158152.2
rs12660679	6	21540396	AL512380.2
rs4841553	8	11549306	BLK
rs5934247	X	6929871	PUDP
rs9642393	7	55177954	EGFR
rs6985267	8	10423308	MSRA
rs2405942	X	9846095	SHROOM2
rs2592551	2	85553008	GGCX
rs17169573	7	16668403	BZW2
rs9947504	18	63920849	SERPINB10
rs17408227	5	31459636	DROSHA
rs3772073	2	160284359	RBMS1
rs543152	18	68290555	AC005909.2
rs2570501	2	103895768	LINC01965
rs4276227	3	32289194	CMTM8
rs635817	1	229815275	LINC01682
rs13099184	3	142455574	ATR
rs769149	11	35510904	PAMR1
rs679958	13	110226660	COL4A1

rs10451920	3	167320151	ZBBX
rs1539214	14	47021176	MDGA2
rs9309559	2	26918647	DPYSL5
rs2140336	4	8423104	ACOX3
rs2296622	1	91696021	TGFBR3
rs7524066	1	91719257	TGFBR3
rs12022777	1	190664850	LINC01720
rs309058	1	98936668	PLPPR5
rs10746488	1	9362248	SPSB1
rs1255135	10	128319373	AL390763.1
rs11051493	12	31628444	AC068774.1
rs12441317	15	98131599	AC022523.3
rs17544073	7	47411974	TNS3
rs524554	3	140470306	CLSTN2
rs10898957	11	74114458	C2CD3
rs130191	22	48722786	TAFA5
rs198197	16	24117066	PRKCB
rs267720	1	30024395	LINC01648
rs11761294	7	91513085	AC079760.1
rs7279730	21	16589224	MIR99AHG
rs11751325	6	24522566	ALDH5A1
rs2034732	2	3834471	DCDC2C
rs9455309	6	71057928	AL096709.1
rs990672	2	229091602	PID1
rs11761294	7	91513085	AC079760.2
rs12619842	2	164088534	AC016766.1
rs1999652	6	6574151	LY86-AS1
rs685098	17	38912682	LASP1
rs12224675	11	11985530	DKK3
rs6765155	3	14687590	C3orf20

rs1468052	6	133688556	TARID
rs5972046	X	36401749	AL606516.1
rs28923193	17	12093379	MAP2K4
rs4960658	7	154983713	PAXIP1
rs12441317	15	98131599	AC022523.1
rs420709	7	102062241	CUX1
rs7432941	3	70213926	MDFIC2
rs1343921	4	89026311	FAM13A
rs1046654	11	94498681	ANKRD49
rs970169	16	58859685	AC106793.1
rs4897633	8	134213081	AC105180.2
rs970169	16	58859685	AC092378.1
rs12445232	16	65101212	CDH11
rs6985267	CHR_HG76_PATCH	9528469	MSRA
rs7432941	3	70213926	SAMMSON
rs4235253	4	8044136	ABLIM2
rs7104359	11	103826689	AP002989.1
rs6497755	16	24775874	TNRC6A
rs4841553	CHR_HG76_PATCH	8403522	AC270286.1
rs11079651	17	66350051	PRKCA
rs4897633	8	134213081	AC105180.1
rs12959390	18	25147011	ZNF521
rs9931092	16	83113320	CDH13
rs1533763	11	76189483	WNT11
rs9921664	16	79015383	WWOX
rs4627704	3	73822774	LINC02005
rs1390203	12	71199261	TSPAN8
rs11211262	1	46255483	RAD54L

Table S3: SNPs found interacting with the Group 2 features in the BC risk prediction task and their associated genes.

Variant	Chromosome	Position start (bp)	Associated gene name
rs3743083	15	78281277	AC090607.4
rs3743083	15	78281277	WDR61
rs3743083	15	78281277	DNAJA4
rs10811186	9	19440125	ACER2
rs9548070	13	37841860	TRPC4
rs4689278	4	5689175	EVC2
rs1078080	10	116064228	GFRA1
rs2955005	8	81706019	ZFAND1
rs1146261	20	36858756	SOGA1
rs1096752	5	77253733	AC022414.1
rs3772073	2	160284359	RBMS1
rs1096752	5	77253733	PDE8B
rs1328710	6	71892861	RIMS1
rs3731714	2	201196097	CASP10
rs10811657	9	22127642	CDKN2B-AS1
rs2592551	2	85553008	GGCX
rs11985853	8	127619279	AC104370.1
rs13052371	21	32106374	LINC00159
rs140519	22	50549633	KLHDC7B
rs6138482	20	25078806	VSX1
rs2515767	12	11825015	ETV6
rs12448631	16	25948481	AC093516.1
rs2272300	12	53193684	ZNF740
rs2272300	12	53193684	ITGB7
rs12441317	15	98131599	AC022523.3
rs10746488	1	9362248	SPSB1
rs198197	16	24117066	PRKCB
rs635817	1	229815275	LINC01682
rs9455309	6	71057928	AL096709.1
rs1999652	6	6574151	LY86-AS1
rs13099184	3	142455574	ATR
rs2923731	15	85048516	PDE8A
rs2034732	2	3834471	DCDC2C
rs3027001	1	159199673	CADM3-AS1
rs2954669	8	144809348	ZNF517
rs2188278	7	106375865	AC004917.1
rs10898957	11	74114458	C2CD3
rs1371736	7	9149856	AC004852.2
rs1468052	6	133688556	TARID
rs11051493	12	31628444	AC068774.1
rs573123	6	149312973	TAB2
rs1327786	9	109784497	PALM2-AKAP2
rs3828191	2	164493392	GRB14
rs1041992	19	41625899	CEACAM4
rs1041992	CHR_HSCHR19_3_CTG3_1	41634241	CEACAM4

rs309058	1	98936668	PLPPR5
rs12441317	15	98131599	AC022523.1
rs12448631	16	25948481	HS3ST4
rs11751325	6	24522566	ALDH5A1
rs4960658	7	154983713	PAXIP1
rs7614	18	54154874	MBD2
rs10899600	11	79166383	TENM4
rs3027001	1	159199673	CADM3
rs13191563	6	69003165	ADGRB3
rs4682664	3	134201065	RYK
rs2709437	2	5983360	SILC1
rs11594610	10	113153224	TCF7L2
rs16831558	1	44036491	AL139220.2
rs11757540	6	151826072	ESR1
rs827423	6	151835062	ESR1
rs11794667	9	16342067	C9orf92
rs1533763	11	76189483	WNT11
rs6757785	2	172431436	ITGA6
rs4627704	3	73822774	LINC02005
rs1343921	4	89026311	FAM13A
rs10513808	3	186938656	ST6GAL1
rs3828057	1	151807701	RORC
rs10847255	12	127011943	LINC02405
rs10495062	1	217631613	SPATA17
rs6531084	2	16378317	AC010880.1
rs1007938	12	26649616	ITPR2
rs10163440	16	77660400	AC092724.1
rs11211262	1	46255483	RAD54L
rs2569475	19	51062718	KLK13
rs4897633	8	134213081	AC105180.2
rs10503929	8	32756465	NRG1
rs10878264	12	65365742	MSRB3
rs12599670	16	83937504	MLYCD
rs1649200	10	121484216	FGFR2
rs12599670	16	83937504	OSGIN1
rs1046654	11	94498681	ANKRD49
rs34729252	6	139361625	AL592429.2
rs12380828	9	82744914	AL162726.3
rs1075010	12	13765407	GRIN2B
rs4897633	8	134213081	AC105180.1
rs12988180	2	200857433	CLK1
rs10847255	12	127011943	LINC02405
rs954811	16	78907874	WWOX
rs7104359	11	103826689	AP002989.1
rs313403	11	103321803	DYNC2H1
